# Diabetic retinopathy in sub-Saharan Africa: meeting the challenges of an emerging epidemic

**DOI:** 10.1186/1741-7015-11-157

**Published:** 2013-07-02

**Authors:** Philip I Burgess, Gerald Msukwa, Nicholas AV Beare

**Affiliations:** 1 Malawi-Liverpool-Wellcome Trust Clinical Research Programme, Queen Elizabeth Central Hospital, College of Medicine, P.O. Box 30096, Chichiri, Blantyre 3, Malawi; 2 Lions First Sight Eye Unit, Queen Elizabeth Central Hospital, Blantyre, Malawi; 3 St Pauls Eye Unit, Royal Liverpool University Hospital, Liverpool, UK; 4 Department of Eye and Vision Sciences, University of Liverpool, Liverpool, UK

**Keywords:** Africa, Diabetic retinopathy, Epidemiology, Health services, Screening

## Abstract

**Background:**

Sub-Saharan Africa faces an epidemic of diabetes. Diabetes causes significant morbidity including visual loss from diabetic retinopathy, which is largely preventable. In this resource-poor setting, health systems are poorly organized to deliver chronic care with multiple system involvement. The specific skills and resources needed to manage diabetic retinopathy are scarce. The costs of inaction for individuals, communities and countries are likely to be high.

**Discussion:**

Screening for and treatment of diabetic retinopathy have been shown to be effective, and cost-effective, in resource-rich settings. In sub-Saharan Africa, clinical services for diabetes need to be expanded with the provision of effective, integrated care, including case-finding and management of diabetic retinopathy. This should be underpinned by a high quality evidence base accounting for differences in diabetes types, resources, patients and society in Africa. Research must address the epidemiology of diabetic retinopathy in Africa, strategies for disease detection and management with laser treatment, and include health economic analyses. Models of care tailored to the local geographic and social context are most likely to be cost effective, and should draw on experience and expertise from other continents. Research into diabetic retinopathy in Africa can drive the political agenda for service development and enable informed prioritization of available health funding at a national level. Effective interventions need to be implemented in the near future to avert a large burden of visual loss from diabetic retinopathy in the continent.

**Summary:**

An increase in visual loss from diabetic retinopathy is inevitable as the diabetes epidemic emerges in sub-Saharan Africa. This could be minimized by the provision of case-finding and laser treatment, but how to do this most effectively in the regional context is not known. Research into the epidemiology, case-finding and laser treatment of diabetic retinopathy in sub-Saharan Africa will highlight a poorly met need, as well as guide the development of services for that need as it expands.

## Background

Sub-Saharan Africa (SSA) faces an epidemic of noncommunicable disease (NCD) driven by urbanization, lifestyle, poor diet, smoking, environmental factors and aging. Increased life expectancy and population growth (in part from successes in combating communicable diseases) add to the NCD burden to society, health services and the individual [1].

The International Diabetes Federation has estimated that the number of adults with diabetes in Africa will double in 20 years, from 12 million in 2010 to 24 million in 2030 [2]. Diabetes causes significant morbidity, disability and early mortality. The diabetes epidemic therefore poses significant health and socioeconomic challenges for a continent simultaneously facing other health challenges including infectious diseases (HIV, tuberculosis and malaria) and high levels of maternal and perinatal disorders and trauma.

Diabetes causes visual impairment (VI) through early-onset cataract and diabetic retinopathy (DR), a progressive disease of the retinal microvasculature. Cataract and DR are the second and sixth leading causes of global VI, respectively [3]. Both are included in the list of nine target diseases of Vision 2020, a joint program of the World Health Organization (WHO) and the International Agency for the Prevention of Blindness. Diabetes damages retinal capillaries through prolonged exposure to hyperglycemia. This leads to loss of supporting pericyte cells and tight junctions between endothelial cells. In turn, this causes leakage from capillaries, resulting in retinal edema; capillary closure; and ischemia. An ischemic retina produces vascular endothelial growth factor (VEGF), which stimulates new vessel growth (proliferative diabetic retinopathy, PDR). An edematous or ischemic retina loses function, and this will reduce vision if the central retina or macular is involved. New vessels are prone to bleeding (vitreous hemorrhage) and the accompanying fibrosis leads to tractional retinal detachment. Thus the sight-threatening manifestations of DR are proliferative retinopathy and diabetic maculopathy, which are both preventable and treatable before vision is lost. The risk of development and progression of retinopathy has been shown in developed economies to be related to glycemic control or HbA1c [4-7], blood pressure [4,8-11] and blood lipid levels [12]. Laser photocoagulation has been shown to be effective at reducing visual loss in patients with PDR [13] and macular edema [14] if timely treatment is performed.

The epidemiology of DR in Africa has been systematically reviewed by our group [15]. This review identified no community-based cross-sectional or cohort studies of DR from SSA on which to base incidence or prevalence estimates in the population with diabetes. A recent population-based survey (n = 4,414) in Nakuru, Kenya, identified a prevalence of ‘any DR’ of 35.9% (95% CI: 29.7, 42.6) and of ‘severe non proliferative DR or PDR’ of 13.9% (95% CI: 10.0, 18.8) in 277 people with diabetes [Bastawrous, personal communication]. Clinic-based studies report a wide range of prevalence, often with higher levels of sight-threatening disease, but these are subject to bias. The proportion of any VI in African populations due to DR is largely unknown. A recent population-based study from Cape Town, South Africa, of visual loss using WHO methods identified DR as the cause of 8% of blindness and 11% of severe visual loss in persons ≥50 years [16]. Population-based surveys of VI usually underestimate DR as a cause. Retinal causes of VI are often recorded together as one category, and are not recorded if there is no fundal view (for example, due to cataract). Rectifiable causes of VI (for example, cataract) are recorded in preference to preventable or untreatable causes [17].

In this article we first review the evidence on detection and management of DR in Africa. We discuss the potential costs and benefits of action on DR within an integrated strategy for diabetes care. Finally we propose that, drawing on experience and expertise from other continents, research into DR in Africa can drive the political agenda for service development.

## Discussion

### Evidence on natural history and management of diabetic retinopathy in an African setting is lacking

#### Determinants of severity and progression

The prevalence and incidence of sight-threatening DR in developed countries have been well documented [18-20]. Associations between systemic factors and the development of sight-threatening DR in these populations are well known [4-12]. By contrast, only two cohort studies have investigated determinants of severity and progression of DR in Africa: a population based study from Mauritius [21] and a relatively small study of patients with type 1 diabetes in South Africa [22]. There may be additional factors which affect DR in SSA, and poor glycemic and blood pressure control may result in rapid progression.

As well as the type 1 and 2 paradigm, additional forms of diabetes are described on the continent including ‘malnutrition-related diabetes’ and ‘atypical ketosis prone diabetes’ [1]. In many African countries, the diet is high in refined carbohydrates (for example, maize meal), something that is difficult to change because of cultural and economic reasons. Population-specific risk factors are also likely to affect the spectrum of pathology encountered. Diabetes increases the incident risk of tuberculosis, with worsened outcomes for both [23,24]. Antiretroviral therapy, and possibly HIV itself, is associated with an increased risk of developing metabolic syndrome and diabetes [24]. However, the effect of these factors, as well as of others including infective diseases and deficient micronutrients, on DR are unknown.

#### Effectiveness of screening and laser treatment

Laser treatment for PDR involves ablation of the peripheral retina with 1,000 to 5,000 spaced burns to reduce the amount of ischemic retina and the VEGF it produces. Laser for diabetic macular edema involves applying the treatment directly to leaking microaneurysms, and gentle laser (up to 300 burns) to the macula, thought to stimulate cellular fluid pumps. Laser treatment cannot be used on vision loss caused by ischemic maculopathy. Effectiveness of laser photocoagulation for DR in the African population is assumed from landmark studies of peripheral retinal laser and macular laser in Europe and North America [13,14]. There is no evidence that the benefits of timely laser treatment will be any less in an African population than they are in developed countries.

There have been scattered reports of successful attempts to screen patients attending diabetes clinics for DR [25-29]. Screening using retinal photography has been piloted [26,27,29]. However, most reports have come from South Africa and these efforts have largely been initiated by local hospitals or by external funding and support. Mumba *et al*. [29] in Tanzania attempted to develop a register of known patients with diabetes. Defining an eligible population is an essential component of a screening program but is another challenge in resource-poor settings. Apart from rudimentary costing [26], no evidence on cost effectiveness of screening programs on the continent is available. The fact that patients present late with diabetes must be considered. Late presentation with established complications, in part due to poor education and large indirect costs of accessing care, may affect the benefits of screening. Targeted case findings may be more appropriate.

### Costs of action and inaction

#### Diabetes and its complications affect the working age population

The costs of diabetes to Africa are significant, and are rising rapidly. Kirigia *et al*. [30] estimated that the total economic cost (direct and indirect) of diabetes in the WHO Africa region in 2000 was US$67 billion: equivalent to US$8,836 per person with diabetes per year. A significant proportion of this figure is accounted for by the opportunity cost of productive time lost due to permanent disability and premature mortality. The cost of complications was excluded from this study and therefore true costs are underestimated.

The burden of diabetes and its complications is borne predominantly by the working age population [31]. DR is the commonest cause of blindness in the working population in the USA and Europe [32]. A disease which reduces the economic activity of this group affects individual, household and national economies. To our knowledge, no study has addressed the specific costs of DR in Africa. This information is critical for policymakers to highlight the importance of introducing cost-effective interventions for primary prevention and subsequently detection and treatment of DR.

#### There is a scarcity of resources for treating DR

The agenda for diabetes care in SSA is dominated at a national level by poorly resourced health services and at a community level by poverty. The International Diabetes Federation has estimated that in 2010 national funding for the care of diabetes in Africa was just US$111 per person [33]. This figure is equivalent, on average, to 7% of national healthcare expenditure but varies widely between countries. In Malawi the total annual per capita expenditure on all healthcare was only US$25 [34]. Opportunity costs will also be lower with the Gross National Income per capita only being $330, but it is clear that current expenditure is a fraction of the cost of the disease. Limited public funding means individual patients and their families may have to spend significant proportions of their income on diabetes treatment. Diabetes care must compete with infective diseases and other healthcare initiatives in terms of political and financial priorities. It does not lend itself to the vertical programs favored by donors. However, political attention is now turning to NCDs, as witnessed by the United Nations high-level meeting on NCD prevention and control, held in September 2011 [35]. Policy makers require evidence-based guidance on resource allocation.

The rise of NCDs in Africa demands not only resource allocation but massive reorganization of health systems. SSA faces a large burden of infectious diseases (HIV, tuberculosis and malaria) and high levels of maternal and perinatal disorders and trauma. Services have traditionally been organized to manage distinct health events (for example, episodes of infection, child birth). NCDs demand effective, integrated multidisciplinary services over a lifetime. This is a significant challenge for health providers as it impacts on all elements of health systems: workforce, facilities, technology and pharmaceuticals as well as leadership and governance. Reductions in microvascular complications from improved glycemic and blood pressure control, as shown in the Diabetes Complications and Control Trial [5] and United Kingdom Prospective Diabetes Study [8], will be maximized if monitoring and medications are consistently available. Similarly, provision of services for detection and management of complications, including DR, must be consistent rather than intermittent.

In many SSA countries, services for detection and management of DR are currently rudimentary and only available in urban settings. There is a significant shortfall of ophthalmologists. A recent survey showed that, while the number of practitioners is increasing in developing countries, the population aged ≥60 years is growing at a greater rate [36,37]. This suggests that the gap between need and supply is widening. Referral pathways between primary and secondary care, and those between diabetic clinics and ophthalmic services, are poorly organized. Patients do not have a family practitioner to help them negotiate services for diabetic complications. Indirect costs of attending hospital are high, and may be perceived as unnecessary when eye disease is not apparent, prior to visual loss.

In Malawi, a country of 15.2 million people [38], there are only seven practicing consultant ophthalmologists. The majority of ophthalmic patients are seen by ophthalmic clinical officers (OCOs) who receive relatively little training in retinal examination and diagnosis. Ophthalmologists and OCOs are overwhelmed by cataract, ocular trauma, infectious disease and pediatric ophthalmology so can devote limited time to retinal disease management. In many SSA countries, lack of equipment is limiting: in Malawi there is no retinal imaging to support diagnoses; there are now two lasers provided by external support. These factors result in the under-development of skills in DR management.

#### Barriers to effective care delivery

Diabetes can be thought of as an index case for NCD healthcare delivery in Africa and developing countries worldwide. It is a chronic disease requiring complex medical management. Its complications affect a variety of body systems; detection and management requires a number of specialist medical services. The education and empowerment of patients is an important part of disease management. In addition to economic factors, a number of barriers exist to delivery of care for diabetes and its complications. WHO has identified the following problems for healthcare delivery in developing countries: lack of organizational structure for chronic disease care; minimal staffing and training provided to healthcare workers; minimal communication with the public to address preventative strategies; non-existence of organized healthcare information systems; and lack of involvement and integration with other community resources [39]. In Africa, these barriers translate into the inadequacies in diabetes care identified by Whiting *et al.* (listed below) [40]. We have identified a number of specific barriers to DR care in Africa which are listed below.

Inadequacies in diabetes care as identified by Whiting *et al*. [40]

1 Poor patient attendance at clinics

2 Low doctor to patient ratio leading to short consultation times and little or no time for patient education

3 Low staff levels including a lack of trained nurses and other health workers

4 Lack of staff training specific to diabetes

5 A lack of systematic evaluation and monitoring of the complications of diabetes

6 Non-existent or inadequate referral systems

7 Poor record keeping

8 Non-existent diabetes multidisciplinary healthcare teams

9 Lack of infrastructure to support services

10 Lack of national policies

Specific barriers to diabetic retinopathy care in the African region

1 Lack of ophthalmologists

2 Low number of ophthalmologists with training and experience in management of DR

3 Low numbers of opticians and OCOs to perform opportunistic screening; commercial opticians are only accessible to the wealthy

4 Lack of training for opticians and OCOs in fundoscopy

5 Inadequate referral systems from primary to secondary care and from medical departments to ophthalmic services

6 Non-existent systematic screening programs

7 Little access to imaging technology including fluorescein angiography and optical coherence tomography

8 Lack of treatment infrastructure including lasers and laser maintenance

9 Lack of national policies and low government priority

### Lessons for sub-Saharan Africa can be learnt from other settings

#### Integration of diabetes services

The evolving African diabetes epidemic necessitates a coordinated response that involves integrating services at a number of levels. At a community level, interventions for the control and management of NCDs are necessary. Several models exist in South Africa, for example the Community Health Intervention Programme and the Woolworths Health Promotion Programme [41]. The effect of these programs on NCD remains under evaluation [41]. Expansion of health center- and also hospital-based diabetes clinics is required on a massive scale. Primary prevention of complications by systemic risk factor management is a priority. Services must be tailored to a resource-poor environment and the local geographic and socioeconomic context. With such a rapid increase in patient numbers, a simplification of some services has been proposed. This might resemble the streamlining of HIV and AIDS services that was necessary to achieve antiretroviral therapy roll out in many states in SSA [42]. Some have advocated a ‘poly pill’ approach to vascular risk factor management in Africa, although this approach has significant drawbacks [43].

Systematic evaluation of complications with robust referral mechanisms to specialist services is required. The coordination of services at a local level aids this process. Provision of medical management of diabetes, patient education, and detection of complications via, for example, screening for DR at a single time and place in the community improve access to care. In resource-poor settings patients may travel long distances to attend health services incurring transport costs and loss of income. The involvement of government agencies is key to long-term service development. Outside funding and expertise may be vital in setting up health systems. However, sustainability and large-scale roll out of services necessitates national policies. Patient organizations are important advocates for services and can help create political momentum.

#### Laser photocoagulation is the mainstay of treatment for diabetic retinopathy and maculopathy

The effectiveness of laser photocoagulation at reducing the likelihood of VI and blindness in patients with PDR [13] and macular edema [14] is well established. Recent evidence demonstrates better outcomes in the short-term from intra-vitreal anti-VEGF agents (injected intra-ocularly) in diabetic maculopathy that has already reduced vision [44]. This topic is the subject of a recent Cochrane review [45]. These agents, which require multiple repeat injections, will be increasingly used in resource-rich economies although with a continued role for laser. At present these agents are prohibitively expensive for widespread use in resource-poor countries (approximately US$800 per injection for the drug alone). However, off-label use of the systemic anti-VEGF, bevacizumab (Avastin) (approximately US$70 per injection), is used in some African tertiary centers on a paying patient basis, an approach supported by the BOLT study [46]. Vitreoretinal surgery has an important role in managing advanced disease. However, published data from this setting is sparse and more research on long-term outcomes and cost effectiveness is required.

Provision of laser services requires substantial initial investment in equipment and training of ophthalmologists. However, equipment upkeep costs are small and there are no on-going drug costs. The inadequacy of retinal training and paucity of referral networks are significant barriers to service development for DR. A number of proposals to confront these issues are listed below. Our clinical and research group has demonstrated that provision of a laser treatment service is feasible in SSA albeit with external support. In Queen Elizabeth Central Hospital, Blantyre, Malawi, set-up costs of equipment and training of ophthalmologists has been funded by an outside agency: the World Diabetes Foundation. OCOs have been trained in the recognition and referral of DR with funding from the same agency.

Proposals to improve retinal training and retinal referral networks in sub-Saharan Africa

1 Increase the number of ophthalmologists trained and working in the region to allow increased sub-specialization

2 Provision of imaging and treatment infrastructure to allow sub-specialty practice

3 Creation of regional centers of excellence in Africa for provision of tertiary retinal care and training

4 Development of retinal research networks: providing funding both for personnel and equipment, facilitating income generation for eye units, setting standards for clinical practice, improving the evidence base for this setting, setting the political agenda and attracting excellent clinicians.

5 Prioritization of sub-specialty development in post-graduate training programs

6 Promotion of partnership arrangements with retinal centers in developed countries to facilitate knowledge and skill sharing

7 Provision of retinal fellowships tailored to developing world trainees in retinal centers in developed countries

8 Use of donor and government funds to minimize costs of such fellowships for trainees on condition of return to practice in country of origin

#### Effectiveness and cost effectiveness of systematic diabetic retinopathy screening in western settings is well established

Various methods of screening for DR are available. Slit-lamp examination by a trained ophthalmologist and retinal photography with grading of retinopathy by accredited graders can be considered the reference standards for disease detection [47]. Examination with the direct ophthalmoscope is less sensitive and specific for DR [47]. The cost effectiveness of DR screening has been excellently reviewed by Jones *et al*. [48]. Systematic screening is cost-effective for sight years preserved compared with no screening. Variation in age of onset of diabetes, glycemic control, sensitivity of the screening test and compliance rates influence the cost-effectiveness of screening programs.

Digital photography with telemedicine links, as used by the English National Screening Programme [49], has the potential to deliver cost-effective, accessible screening to rural and remote populations. Unfortunately, fundus cameras remain prohibitively expensive. Development of lower cost solutions could radically alter the landscape for DR care in Africa; research into appropriate technologies is needed in addition to studies of disease epidemiology and clinical trials of detection and management strategies. Optimum screening intervals have yet to be defined and research on targeted screening based on disease risk factors is on-going.

#### In Europe, sound evidence has driven service development

DR is the most common cause of blindness in the working age population in the USA and Europe [32]. Landmark epidemiological studies have demonstrated prevalence, incidence and progression of the disease in the UK [50], Europe [51,52] and North America [53]. Studies including Diabetes Complications and Control Trial [5], United Kingdom Prospective Diabetes Study [8], and the Action to Control Cardiovascular Risk in Diabetes trial [54] have shown that control of systemic risk factors can prevent the microvascular complications of diabetes including retinopathy. Tight glycemic control also reduces incidence of cataract [55]. The effectiveness and cost effectiveness of systematic screening programs for DR has been demonstrated [56]. This strong evidence base has driven the political agenda for service development for diabetes and its complications. Further high-quality research on the epidemiology of DR in Africa, and on the effectiveness and cost effectiveness of DR care models tailored to local needs is necessary. This evidence can effect change in national policies which will transform services.

#### Lessons from other resource poor settings

The Indian subcontinent has the greatest number of individuals with diabetes globally [2]. India and surrounding states face many of the same barriers to DR care recognized in Africa: lack of resources and trained manpower to screen and treat the large number of people with DR; unequal distribution of resources between urban and rural areas; and populations with high levels of poverty and poor education many of whom are remote from health services.

A variety of screening and treatment models have been described in India, many utilizing advances in information, medical and communication technology to reduce inequalities in service delivery. Examples include the Aravind Teleophthalmology Network and the Sankara Netralaya Teleophthalmology Project [57]. Economic modeling analysis has been performed on the latter: the rural teleophthalmology program was cost effective compared to no screening [58]. From a health provider perspective, screening at up to 2-year intervals was cost effective while from a societal perspective screening once every 5 years was cost effective [58]. Sustainable models of screening and treatment for DR in Africa could be modeled on those already operational in India.

## Summary

Improvement of services for people with diabetes and its complications is an urgent priority for Africa. The cost of inaction for individuals, communities and countries is likely to be high. The first priorities for diabetes care delivery must be adequate disease detection and appropriate medical management: primary prevention of complications. However, detection and management of diabetes complications including DR within effective integrated diabetes services in an African setting is feasible. Expansion of clinical services requires a shift in national health priorities. A much better evidence base is needed to effect this change. Understanding the scale of the problem and areas where intervention is required will enable informed prioritization of available health funding at a national level.

There is a pressing need for high-quality research into the epidemiology of DR in Africa. The research agenda must also address strategies for disease detection and management and include health economic analyses. Models of care tailored to the local geographic and social context are most likely to be cost effective. Drawing on experience and expertise from other continents will aid design of clinical trials and, in turn, service development. We recognize the complex challenges inherent in health-care provision in SSA. However, effective interventions need to be implemented in the near future to avert a large burden of visual loss from DR on the continent.

## Abbreviations

DR: Diabetic retinopathy; NCD: Noncommunicable disease; OCO: Ophthalmic clinical officers; PDR: Proliferative diabetic retinopathy; SSA: Sub-Saharan Africa; VEGF: Vascular endothelial growth factor; VI: Visual impairment; WHO: World Health Organization.

## Competing interests

The authors declare that they have no competing interests.

## Authors’ contributions

PB drafted the manuscript. GM critically revised manuscript. NB participated in the design of the article and critically revised the manuscript. All authors read and approved the final manuscript.

## Pre-publication history

The pre-publication history for this paper can be accessed here:

http://www.biomedcentral.com/1741-7015/11/157/prepub
